# Meta-Analysis of Comparative Trials Evaluating a Prophylactic Single-Use Negative Pressure Wound Therapy System for the Prevention of Surgical Site Complications

**DOI:** 10.1089/sur.2017.156

**Published:** 2017-10-01

**Authors:** Vicki Strugala, Robin Martin

**Affiliations:** Advanced Wound Management, Clinical, Scientific and Medical Affairs, Smith & Nephew plc, Hull, United Kingdom.

**Keywords:** postoperative complication, prophylaxis, surgical site infection, wound infection, wound management

## Abstract

***Background:*** We report the first meta-analysis on the impact of prophylactic use of a specific design of negative pressure wound therapy (NPWT) device on surgical site complications.

***Methods:*** Articles were identified in which the specific single-use NPWT device (PICO^⋄^, Smith & Nephew) was compared with standard care for surgical site infection (SSI), dehiscence, or length of stay (LOS). Risk ratio (RR) ±95% confidence interval (CI) (SSI; dehiscence) or mean difference in LOS ±95% CI was calculated using RevMan v5.3.

***Results:*** There were 1863 patients (2202 incisions) represented by 16 articles. Among 10 randomized studies, there was a significant reduction in SSI rate of 51% from 9.7% to 4.8% with NPWT intervention (RR 0.49 [95% CI 0.34–0.69] p < 0.0001). There were six observational studies assessing reduction in SSI rate of 67% from 22.5% to 7.4% with NPWT (RR 0. 32 [95% CI 0.18–0.55] p < 0.0001). Combining all 16 studies, there was a significant reduction in SSI of 58% from 12.5% to 5.2% with NPWT (RR 0.43 [95% CI 0.32–0.57] p < 0.0001). Similar effects were seen irrespective of the kind of surgery (orthopedic, abdominal, colorectal, or cesarean section), although the numbers needed to treat (NNT) were lower in operations with higher frequencies of complications. There was a significant reduction in dehiscence from 17.4% to 12.8% with NPWT (RR 0.71 [95% CI 0.54–0.92] p < 0.01). The mean reduction in hospital LOS by NPWT was also significant (−0.47 days [95% CI −0.71 to −0.23] p < 0.0001).

***Conclusions:*** The significant reduction in SSI, wound dehiscence, and LOS on the basis of pooled data from 16 studies shows a benefit of the PICO single-use NPWT system compared with standard care in closed surgical incisions.

Improving outcomes for patients undergoing surgical procedures by reducing rates of surgical site complications (SSC) could have a significant impact on patients' lives and societal and healthcare costs [1]. Complications that may affect closed surgical incisions and ultimately delay healing include surgical site infection (SSI), dehiscence, seroma, and hematoma, and these may result in poor quality or abnormal scar formation. The Centers for Disease Control and Prevention (CDC) definitions of SSIs are widely used for surveillance and research purposes. They classify SSIs as superficial incisional, deep incisional, or organ/space and are applicable to all types of operations [2].

Prevention of SSCs is complex because of the interactions of patient-related, environmental, and surgical factors that may be involved. Risk factors may occur at multiple points during the pre-operative, operative, and post-operative phases of surgical procedures. Efforts to reduce SSI often include a limited number of selected interventions that are grouped together in a “care bundle” [3], but compliance with a care bundle is not assured [4].

Negative pressure wound therapy (NPWT) is emerging as a promising technology as a preventative intervention and is beginning to be advocated within care bundles in a variety of closed surgical incisions to reduce SSC and specifically SSI [1,5–7]. The mode of action of NPWT on a closed surgical incision is notably different from the mode of action in open wounds. The combined effects of reduced lateral tension, improved lymphatic clearance, and reduction in hematoma and seroma, as demonstrated in studies of NPWT on closed surgical incisions, may contribute to faster and stronger healing with reduced risk of infection and dehiscence [6]. Although conventional wisdom might suggest that the NPWT dressing should be targeted just to the sutured incision, there is a developing awareness that the lateral extent of operation may be important and that application of NPWT across a wider surface area (“the zone of injury”) may be a better clinical strategy [1,6,8], because NPWT can act to reduce: edema, excess interstitial fluid, and venous stasis and improve the general condition of the adjacent tissue [9–11].

There is a growing body of clinical evidence in which prophylactic NPWT has been investigated to reduce the risk of SSC in controlled studies, and a number of meta-analysis have been performed on this data [5,12–16]. It must be stressed that these previous meta-analyses include a diverse mix of traditional NPWT and single-use NPWT devices operating at varying levels of negative pressure and mixed surgical indications, which may introduce heterogeneity into meta-analysis.

## Aim

**FIG. 1.**
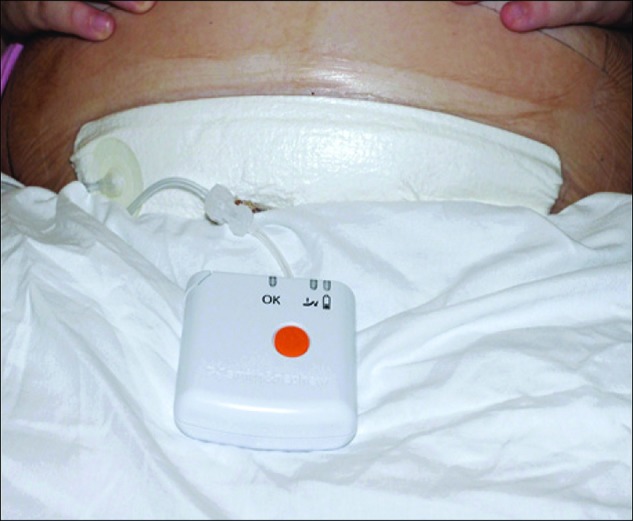
The PICO^⋄^ single use negative pressure wound therapy in position on a patient with a cesarean section closed surgical incision. PICO (Smith & Nephew) is a small, lightweight, ultra-portable, negative pressure system that consists of a dressing, supplied with a small negative pressure pump powered by two AA batteries. The pump is disposable after seven days. The PICO system produces negative pressure at −80 mm Hg continuously, and therapy can be started or paused using the single orange button [53]. Used with permission from Bullogh and Burns [23].

We report the first meta-analysis on SSC occurrence using a specific single-use or disposable NPWT device (PICO^⋄^ single-use NPWT system, Smith & Nephew, Hull, UK) that operates only at −80 mm Hg with the aim of reducing two key variables that are responsible for heterogeneity when different devices are combined. The influence of this specific NPWT device on the rate of SSI, dehiscence, and length of stay (LOS) has been analyzed in comparison with standard care across a number of surgical indications. Disposable or single-use NPWT devices are particularly attractive for preventative use [6]. Figure 1 shows the PICO device in use.

## Methods

This systematic review and meta-analysis is reported in accordance with the Preferred Reporting Items for Systematic reviews and Meta-Analyses (PRISMA) statement [17].

### Eligibility criteria

All known clinical studies comparing single-use NPWT with standard care (any non-NPWT dressing) applied post-operatively on a closed surgical incision were included. The intervention was required to be the PICO single-use NPWT system, which is a small canister-free device that deals with exudate by absorption and evaporation using a unique dressing technology. It delivers −80 mm Hg negative pressure (nominal) for seven days.

Participants of any age and undergoing any type of operation were eligible. There were no restrictions on the inclusion or exclusion criteria with regard to risk factors for complications. Studies had to report either SSI with at least 21 days follow-up, wound dehiscence occurrence, or the LOS (days). Eligible clinical studies could be randomized controlled trials (RCT), retrospective or prospective observational studies, and be of any sample size. No publication restrictions were imposed. Published abstracts or PhD thesis with sufficient information to extract mean and variance data were included. No valid study was excluded. Studies that were not in English, participants were not human, or case reports, case-series, letters, commentaries, notes, or editorials were excluded. Whenever multiple publications of the same data existed, only the most inclusive, comprehensive, and recent one was included.

### Search strategy

A database of all publications in which PICO single-use NPWT was mentioned was compiled. The search strategy was:

• PubMed search (search terms: Negative pressure wound therapy [Title/Abstract] OR Pico [Title/Abstract] OR Renasys [Title/Abstract] OR VAC [Title/Abstract] OR prevena [Title/Abstract] OR NPWT [Title/Abstract] OR topical negative pressure [Title/Abstract] OR incisional NPWT [Title/Abstract]).
• Review of content from key wound conferences or surgical speciality conferences (e.g. EWMA, WUWHS, SAWC, Wounds UK).
• Intelligence from clinicaltrials.gov registry or country equivalents (search terms: PICO^⋄^ AND NPWT OR NPWT) with extraction of study information, including the title, brief description, conditions, interventions, locations and principal investigator.

This systematic review and meta-analysis were based on that PICO^⋄^ database and search terms listed from 2011 up to March 31, 2017. Review of the publications that met the eligibility criteria was performed by two independent reviewers (VS and RM).

### Data extraction

Data were extracted on the number of patients (or more precisely, the number of incisions), type of surgical procedure, duration of prophylactic NPWT, clinical end-points, and frequency of those outcomes (SSI or dehiscence). The mean (± SD [standard deviation]) LOS was also extracted (or derived by calculation from other reported averages and variance). The type of study was noted as either RCT (including interim or pilot) or observational for non-randomized comparative studies.

### Data analysis

Meta-analysis was performed using a fixed effects model in Review Manager (RevMan) software Version 5.3. (Copenhagen: The Nordic Cochrane Centre, The Cochrane Collaboration, 2014). Dichotomous data were analyzed using Mantel-Haenszel (M-H) [18] to calculate risk ratio (RR) ±95% confidence interval (CI). For continuous data, mean (SD) was entered and was analyzed by Inverse Variance to calculate mean difference ±95% CI. Level of heterogeneity across studies was noted by the I_2_ statistic, and random effects meta-analyses modeling was also reported [19].

For the SSI studies, the data were analyzed as (1) RCTs only, (2) observational studies only, (3) all studies combined (overall). Subgroup meta-analyses stratified by type of surgical procedure were performed subsequently. Number needed to treat (NNT) to avoid a complication was calculated. Data are presented as forest plots, and study bias evaluation was presented as a funnel plot.

## Results

### Study selection

**FIG. 2.**
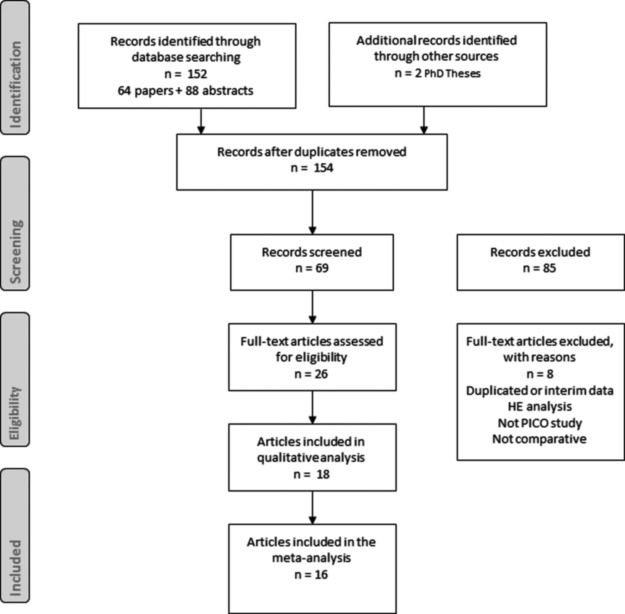
The Preferred Reporting Items for Systematic reviews and Meta-Analyses flowchart showing selection of articles for the meta-analysis.

On the basis of a review of a comprehensive database of 64 articles, 88 posters/abstracts, and one PhD thesis, we selected 26 articles for full text review (Fig. 2 shows the flow chart of the selection of articles for the meta-analysis). After exclusion of eight articles [20–27], finally 18 articles in which PICO was compared with standard care were used in the final qualitative review [8,28–44]. Two articles did not go on to the quantitative meta-analysis because of the SSC end-points not meeting the selection criteria for analysis [8,37]. One article described two separate patient populations (breast and colorectal surgery) that were assessed and analyzed separately [40].

### Study characteristics

All studies used the PICO single-use NPWT system. In the quantitative meta-analysis, there were 16 articles (1863 patients) comprising 10 RCTs reported in 10 articles (including 1734 incisions) and seven observational studies reported in six articles (including 468 incisions). The characteristics of each included study are displayed in Table 1.

**Table 1.** Characteristics of Studies Included in Quantitative Review

*Reference*	*Type of surgical procedure*	*Study design*	*Specific risk factor*	*Duration of PICO^⋄^ treatment*	*Outcome measures (day measured); meta-analysis outcomes in bold*
Adogwa et al 2014 [28]	Spinal surgical procedure for thoracolumbar fusion	Retrospective		3 d	**SSI** (30 d) **Dehiscence** (90 d) SSI (90 d) Readmission rate (30 d) Return to operating theater
Chayboyer et al 2014 [29]	C-section	RCT—pilot	BMI ≥30	4 d	**SSI** (30 d) Type of SSI (30 d) Other wound complications (30 d) **LOS** Readmission (28 d)
Galiano et al 2014 [30]^a^	Breast reduction mammoplasty	RCT—multicenter bilateral study		7–14 d	All complications (21 d) Delayed healing (7 days and 10 d) **Dehiscence** (21 d) **SSI** (21 d) Scar quality
Gillespie et al 2015 [31]	Primary hip arthroplasty	RCT—pilot		5 d	**SSI** (42 d)^b^ SSI indicators (42 d) Wound complications (42 d) **LOS** Readmission
Hasselmann et al 2015 [32]	Vascular surgical procedure	RCT—interim partially bilateral study	Groin incision,	7 d	**SSI** (90) Wound complications (90 d)
Hester et al 2015 [33]	Revision hip or knee arthroplasty	Retrospective		7 d	**SSI** (42 d requiring operation or antibiotics) Dressing related complications
Holt & Murphy 2015 [34]	Breast therapeutic mammoplasty and symmetrizing reduction	Prospective Bilateral study		6 d	**Dehiscence** (6 d and 12 d) Healing time
Hyldig 2016 [35]	C-section	RCT—interim	BMI ≥30	5 d	**SSI** (30 d requiring antibiotic treatment) **Dehiscence** Exudate
Karlakki et al 2016 [36]	Primary hip or knee arthroplasty	RCT		7 d	**LOS** Surgical site complications (including **SSI)** (42 d)^c^ Exudate Dressing changes
Matsumoto and Parekh 2015 [38]	Primary ankle arthroplasty	Retrospective		6 d	Wound healing problems (21 d) **SSI** (30 d)
O'Leary et al 2016 [30]	Laparotomy for colorectal or gynecological operation	RCT		4 d	**SSI** (30 d) SSI (4 d) **LOS** Patient satisfaction
Pellino et al 2014 [40]	Colorectal operation and breast surgical procedure	Prospective		7 d	**SSI** (30 d) ASEPSIS score Seroma **LOS**
Selvaggi et al 2014[41]	Colorectal operation	Prospective	Crohn disease (structuring)	7 d	**SSI** (30 d) ASEPSIS score Seroma **LOS**
Tuuli et al 2017 [42]	C-section	RCT—pilot	BMI ≥30	4 d	SSC (30 ) **SSI** (30 d) **Dehiscence (**30 d) Seroma (30 d) Hematoma (30 d) Pain score (2 d)
Uchino et al 2016 [43]	Ileostomy closure by purse string suture	RCT—feasibility		14 d^d^	Time to complete wound healing **SSI** (30 d)
Witt-Majchrzak et al 2014 [44]	Coronary artery bypass graft (sternotomy)	RCT		5–6 d	SSC **SSI** (30 d)

^a^ Full manuscript of this abstract/poster submitted.

^b^ Per protocol method of analysis calculated as an ITT NPWT; patient was wrongly given standard care and developed an SSI. Published ITT values = 2/35 (5.7%) NPWT; 3/35 (8.6%) standard care.

^c^ SSC rates provided but listed complications in detail such that SSI rate can be calculated. Published SSC rates = 2/102 (2.0%) NPWT; 9/107 (8.4%) standard care.

^d^ PICO^⋄^ applied 24 h after operation.

SSI = surgical site infection; RCT = randomized controlled trial; BMI = body mass index; LOS = length of stay; SSC = surgical site complication; ITT = intention to treat; NPWT = negative pressure wound therapy.

Articles not included in quantitative assessment [8,37].

### Synthesis of results

#### SSI

The rate of SSI was reported in 15 articles representing 16 studies and 1839 patients and comprising 2154 incisions [28–33,35,36,38–44]; the summary statistics of the analysis are shown in Table 2.

**Table 2.** Summary of Surgical Site Complications Clinical Outcomes of PICO^⋄^ Single-Use Negative Pressure Wound Therapy Compared with Standard Care

	*Risk with PICO^⋄^*	*Risk with standard care*	*Relative reduction*	Number patients^*^	*Risk ratio (95% CI)*	*I_2_*	*NNT*
SSI–RCT	4.8%	9.7%	51%	1734	0.49 (0.34 to 0.69)	9%	20
SSI–Observational	7.4%	22.5%	67%	420	0.32 (0.18 to 0.55)	0%	7
SSI–Overall	5.2%	12.5%	58%	2154	0.43 (0.32 to 0.57)	7%	14
Dehiscence–Overall	12.8%	17.4%	26%	1291	0.71 (0.54 to 0.92)	0%	22

^*^ = incisions

CI = confidence interval; NNT = number needed to treat; SSI = surgical site infection; RCT = randomized controlled trial.

On the basis of the 10 RCTs of varying patient numbers, there was a significant reduction in the relative risk of SSI with PICO^⋄^ treatment compared with standard care from 9.7% to 4.8%, which represents an absolute reduction of 4.9% and a relative reduction of 51% (fixed effects RR 0.49 [95% CI 0.34–0.69] p < 0.00001).

In comparison, there were six studies that assessed the SSI rate with non-randomized observational methodology, and these also noted a significant reduction in relative risk of SSI with PICO treatment compared with standard care—22.5% to 7.4%—which is a 15.1% absolute reduction and 67% relative reduction (fixed effects RR 0.32 [95% CI 0.18–055] p < 0.00001).

Overall, combining RCT and observational studies, the rate of SSI was 5.2**%** (54/1037) with PICO single-use NPWT treatment compared with 12.5**%** (140/1117) in the standard care group equating to an absolute reduction of 7.3% and a relative reduction of 58% (fixed effects RR 0.43 [95% CI 0.32–0.57] p < 0.0001).

**FIG. 3.**
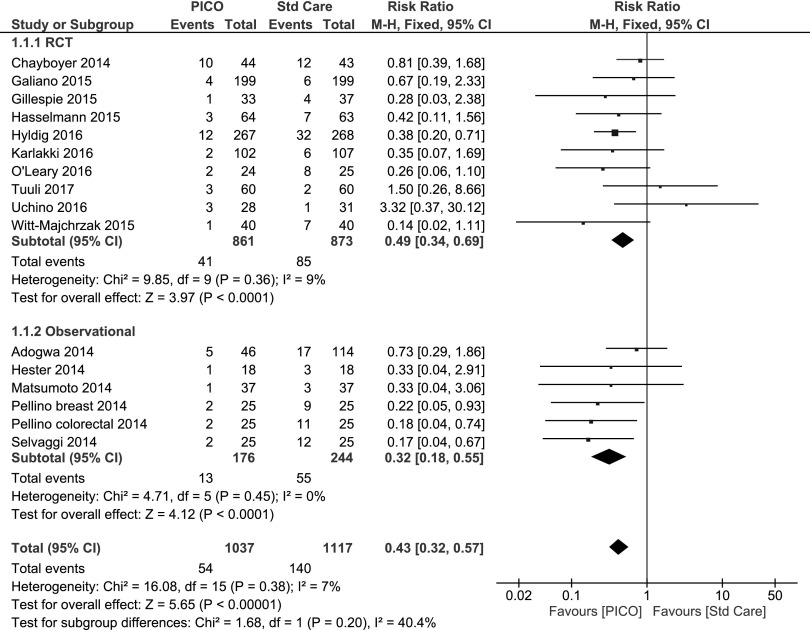
Forest plot of the comparison of PICO single-use negative pressure wound therapy compared with standard care on surgical site infection outcome by randomized controlled trial (RCT), observational, and overall. Random effects model risk ratio 0.45 (95% confidence interval 0.32–0.62) overall.

The details of each individual study and the RCT, observation, and overall data analysis (as a forest plot) are shown in Figure 3. The optimum estimate of the effect was obtained using the strong RCT evidence because there were more studies.

A subgroup analysis was performed on the basis of individual surgical specialities if three or more studies existed (Table 3). Significant reductions in SSI risk compared with standard care was seen in orthopedic surgical procedures (RR 0.48 [95% CI 0.25–0.94] p = 0.03), abdominal operations (RR 0.44 [95% CI 0.30–0.64] p < 0.0001), colorectal surgical procedures (RR 0.29 [95% CI 0.15–0.57] p = 0.0004), and cesarean section (RR 0.53 [95% CI 0.33–0.84] p = 0.007). The degree of relative reduction in the SSI rate was similar (49%–71%) irrespective of the inherent SSI rate of the surgical procedure; however, the NNT is lower in the operations with the higher frequencies of complication.

**Table 3.** Summary of Surgical Site Infection Clinical Outcome of PICO^⋄^ Single-Use Negative Pressure Wound Therapy Compared with Standard Care according to Surgical Indication

*Surgical procedure*	*Risk with PICO^⋄^*	*Risk with standard care*	*Relative reduction*	*Number studies*	*Risk ratio (95% CI)*	*NNT*
Orthopedic - Reconstruction	4.2%	10.5%	60%	5	0.48 (0.25 to 0.93)	16
Colorectal	8.8%	30.2%	71%	4	0.29 (0.15 to 0.57)	5
Cesarean section	6.7%	13.2%	49%	3	0.53 (0.33 to 0.84)	15
Abdominal (colorectal + C-section)	7.2%	16.4%	56%	7	0.44 (0.30 to 0.64)	11

CI = confidence interval; NNT = number needed to treat.

#### Dehiscence

**FIG. 4.**
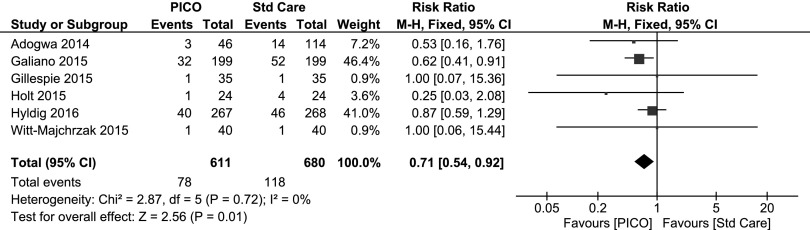
Forest plot of the comparison of PICO^⋄^ single-use negative pressure wound therapy compared with standard care on dehiscence outcome. Random effects model RR 0.72 (95% CI 0.55–0.93).

Rate of wound dehiscence was reported in six articles and 1068 patients, comprising 1291 incisions [28,30,31,34,35,44]. The rate of dehiscence was 12.8**%** (78/611) with PICO single-use NPWT treatment compared with 17.4**%** (118/680) in the standard care group (Table 2) relating to an absolute reduction of 4.6% and a relative reduction of 26.4%. There was a significant reduction in the relative risk of dehiscence with PICO treatment compared with standard care (fixed effects RR 0.71 [95% CI 0.54–0.92] p = 0.01). The details of each individual study and the overall data analysis (as a forest plot) are shown in Figure 4.

#### LOS

**FIG. 5.**
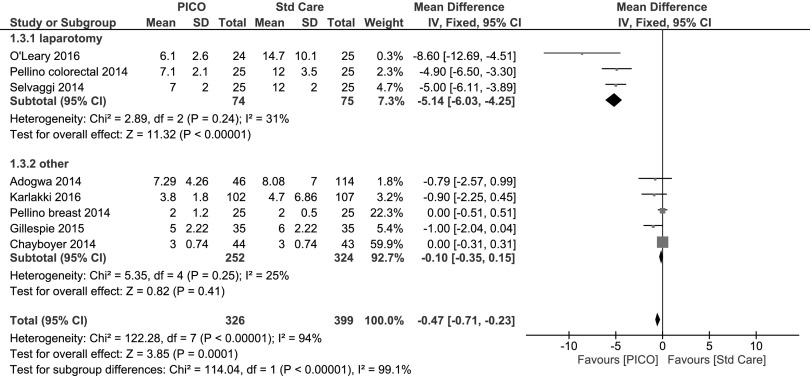
Forest plot of the comparison of PICO^⋄^ single-use negative pressure wound therapy compared with standard care on length of stay with subgroup analysis. Random effects model for all surgical procedures −2.15 (95% CI −3.46 to −0.84).

The LOS was reported in eight studies representing 725 patients [28,29,31,36,39–41]. There was a significant mean difference in LOS from PICO single-use NPWT treatment compared with standard care (−0.47 days [95% CI −0.71 to −0.23] p < 0.0001). The detail of each individual study and the overall data and subgroup analysis (as a forest plot) is shown in Figure 5. A subgroup analysis was performed on the basis of surgical speciality, and mean difference in LOS was −5.1 days in colorectal/laparotomy surgical procedures [39–41] but only −0.1 days in all other operations [28,29,31,36,40].

### Publication bias

**FIG. 6.**
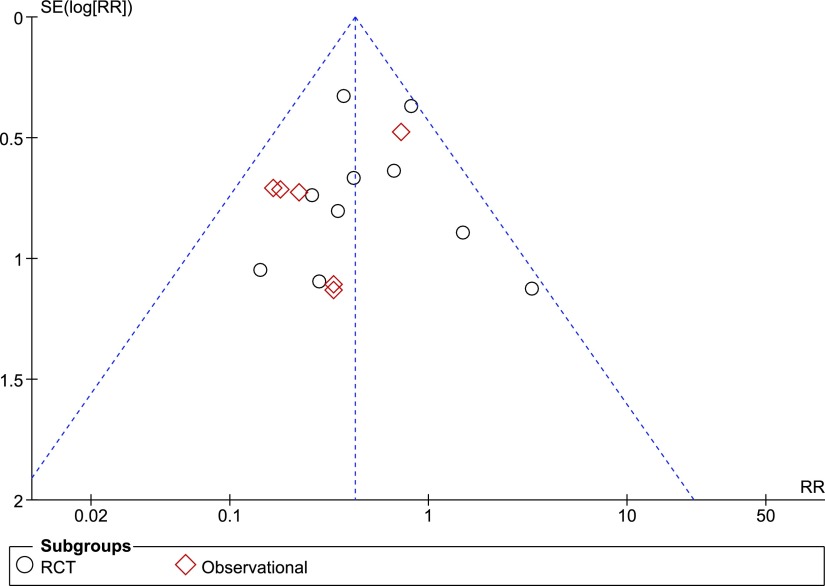
Funnel plot of comparison PICO^⋄^ single-use negative pressure wound therapy compared with standard care on surgical site infection outcomes. SE = standard error; RR = risk ratio; RCT = randomized controlled trial.

An assessment of the publication bias on the basis of the 15 articles included for an SSI outcome is shown in the funnel plot (Fig. 6). The distribution of studies is symmetric, suggesting no publication bias. because both large and small (pilot/ feasibility) studies regardless of the outcome are being published.

## Discussion

On the basis of 16 published clinical papers in which the PICO single-use NPWT device was compared with standard care, there was a clear and significant benefit in favor of prophylactic NPWT to reduce SSI, wound dehiscence, and LOS when the device was applied on a closed surgical incision. The SSCs may delay healing and result in considerable morbidity, death, and socioeconomic costs, and up to 60% are considered to be preventable [1]. There is now also an argument that SSC, and SSI in particular, could have a detrimental effect on oncologic survival [45–47]. The PICO single-use NPWT is shown to be an intervention that reduces SSC and therefore the socioeconomic sequelae.

This is the first comprehensive meta-analysis that has assessed the impact of a specific design of NPWT system on SSCs. By limiting the meta-analysis to a single brand of NPWT with a fixed level of negative pressure (−80 mm Hg), some heterogeneity was reduced. There still remains a heterogeneous mix of surgical indications and patient profiles but the sub-group analysis confirms that the benefit of PICO on SSI remains with stratification. On the basis of the I_2_ statistic, it confirms the homogenous study population and strengthens the conclusions that can be made (I_2_ = 9% for 10 RCTs, 0% for six observational studies, and 7% for the combined 16 studies). Other reviews and meta-analyses have included a diverse mix of traditional NPWT and single-use NPWT devices, varying levels of negative pressure, and varying materials/fillers. There was also calculation of an effect on dehiscence again with a homogenous study population (I_2_ = 0%). On the basis of a higher level of heterogeneity in the LOS data (I_2_ = 94% when 8 studies combined), however, the conclusions for this specific analysis need to be downgraded but show it is worth investigating LOS further.

De Vries et al [15] published recently the supporting meta-analysis data for prophylactic use of NPWT on SSI risk as part of the World Health Organization recommendations for SSI prevention [7]. They stratified their meta-analysis by RCT and also by comparative observational studies, like our study. They reviewed six RCTs and reported a reduction in SSI with RR 0.56. Their observational study analysis comprised 15 studies, and they noted decrease in SSI rate with RR 0.29.

The PICO-only data in this present analysis confirm that this specific design operating at −80 mm Hg is proven to reduce the incidence of SSI across several surgical indications. This echoes the W.H.O. conditional recommendations that suggest *the use of prophylactic NPWT on primarily closed surgical incisions in high-risk wounds for the purpose of preventing SSI, taking resources into account* [7]. It also supports consensus findings that advocate the prophylactic use of NPWT on closed surgical incisions when either patient or procedure risk factors exist [1].

To support the widespread adoption of the use of prophylactic NPWT, of course there needs to be supporting health economic evidence to show that investing in prevention delivers a net benefit for patients and healthcare systems. These studies are now emerging [27,48]. At an approximate cost of $225 for a week of preventative NPWT (April 2017) and modest numbers for NNT, PICO single-use NPWT may prove to be a cost-effective tool in the reduction of surgical complications.

This is a fast moving field. Even with this specific design of single-use NPWT device, new studies are being published regularly, and there are many studies ongoing as noted by published protocols [49–52] or listing on public registries, such as clinicaltrials.gov. Evidence is broader for SSI than for dehiscence or LOS, and it has not yet been possible to individually analyze for superficial, deep, or organ space SSI.

## Conclusion

This is the first comprehensive meta-analysis that has assessed the benefit of a specific brand of single-use NPWT on SSCs. It showed a clear and significant reduction in SSIs, wound dehiscence, and LOS by application of the PICO single-use NPWT system used prophylactically on a closed surgical incision when compared with standard care.
